# Hopf Bifurcation and Vibration Control for a Thrust Magnetic Bearing with Variable Load Mass

**DOI:** 10.3390/s18072212

**Published:** 2018-07-10

**Authors:** Lingling Zhang

**Affiliations:** 1 Institute of Applied Mathematics and Intelligent Information Processing, Hunan Women’s University, Changsha 410004, China; Lingling.Zhang@uts.edu.au or z002005@163.com or linglingmath@gmail.com; 2 School of Mechanical and Mechatronic Engineering, University of Technology Sydney, Ultimo, NSW 2007, Australia

**Keywords:** thrust magnetic bearing, hopf bifurcation, vibration control, variable mass, linear adaptive control

## Abstract

In the working process, the load mass of the thrust magnetic bearing has a significant change. If the load mass changes greatly, the original fixed control parameters cannot ensure that the system is in the optimal stable suspension state, and the performance of the system will become worse or even self-excited. Firstly, a single freedom degree of the suspension control system model is established, and the critical condition of the system is analyzed when a self-excited oscillation occurs. Then, a linear adaptive control law is proposed for the system with variable parameters, which can tolerate the wide range of load mass. The simulation results show that the adaptive control law can keep the stability of the system when the load mass varies in a large range and avoid the self-excited vibration.

## 1. Introduction

In recent years, magnetic bearing has been developed and commercialized due to its advantages of no mechanical contact and no need of lubrication. In all kinds of active magnetic bearings, the thrust magnetic bearing can adjust the axial displacement of the rotor shaft, and is usually used in the thrust direction active suspension control [1]. For the thrust magnetic bearing, the load mass often changes greatly during the working process.When the load mass of the thrust bearing changes, the corresponding parameters of the system will change, and the balance point of the system will also deviate from the original balance point [2,3]. At this time, the original fixed control parameters will not ensure that the system is in the optimal stable suspension state, and the performance of the system will become worse or even self excited [4].

Self-excited vibration is often encountered in the magnetic levitation control system and is one of the most difficult problems to be solved [1,5,6]. On the one hand, the self-excited vibration aggravates the workload of control system and increases the energy consumption of the system. On the other hand, the self-excited vibration makes the performance of control system deteriorate and directly affects the stability of the whole system. Therefore, the control stability of the thrust magnetic bearing must be solved when the load mass is changed in a large range.

The study shows that the self-excited vibration of the magnetic levitation thrust bearing is closely related to the bifurcation of the system equation [1,7,8]. In fact, the thrust magnetic bearing is a typical open-loop unstable nonlinear system. In engineering, the nonlinear system is often linearized at its equilibrium point, and then a corresponding PID controller is designed for the nonlinear system. The practice shows that the stability range of the linear controller designed for the nonlinear system is limited. When the change of the equilibrium point of the nonlinear system is relatively small, the designed linear controller is usually effective. Once a certain parameter of the system changes greatly, such as the parameter of the load mass, the balance point of the system will also shift considerably. Then, the above linear controller will not adapt to the new state [2,8,9].

Many researchers have considered the effect on the stability and bifurcation of the active thrust magnetic bearing rotor systems [10,11]. The model is reduced by a component mode synthesis method, which can conveniently account for nonlinear magnetic forces and moments of the bearing. Then, the system equations are obtained by combining the equations of the reduced mechanical system and the equations of the decentralized PID controllers. The local stability and bifurcation behaviors of periodic motions are obtained by using Floquet theory.

In fact, there are some common ways to solve this problem for this nonlinear system. The feedback linearization method is mainly used in the design of nonlinear system controller, and it has received extensive attention in recent years [12,13,14]. Because the feedback linearization method utilizes all the nonlinear descriptions of maglev system, its control performance will not change with the change of system working point in a large range. The disadvantage of this method is that the whole states of the system are need to be measured, and in practical engineering practice, it is often difficult to meet. In addition, when the system parameters are uncertain, the performance of the system with the feedback linearization control method cannot be guaranteed [14].

In view of the above problems, this paper focuses on the linear adaptive control method of magnetic levitation thrust bearing when the load mass varies greatly. First, the suspension control system model of the thrust magnetic bearing is given, and then the characteristics of the system are analyzed. An adaptive control law considering the variable parameters of load mass is designed. Finally, the effectiveness of the above method is verified by simulation.

## 2. Linear Stability Analysis and Existence of Hopf Bifurcation

**Figure 1:**
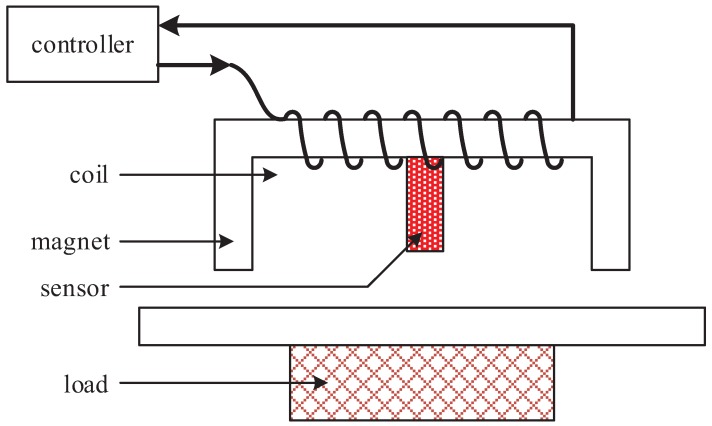
The suspension control system of the electromagnetic thrust bearing.

The suspension control system of the electromagnetic thrust bearing is shown in Figure 1. It is composed of the electromagnet, the coils, the suspension controller, the displacement sensor, the load and so on.

As shown in Figure 1, the sensor can detect in real time the displacement between the electromagnet and the load platform, and transmit the displacement signal to the controller. The controller can calculate the appropriate control value and then provide the appropriate current to the electromagnet coil winding. The magnet will generate enough electromagnetic force to overcome the gravity of the load and achieve a stable suspension state.

The relationship about the electromagnetic force generated by the electromagnet, the current and the magnetic gap is [6,15,16]

(1) $$F=\frac{u_{0}N^{2}Ai^{2}}{4z^{2}}=k\frac{i^{2}}{z^{2}},$$

where $u_{0}$ is the magnetic permeability in vacuum, *N* is the number of turns of coil, *A* is the pole area, *i* is the current and *z* is the length of the magnetic gap.

The dynamical equation of the load is

(2) $$ma=mg-F+f_{d},$$

where *m* is the load mass, *g* is the acceleration of gravity, *a* is the gravity acceleration in the vertical direction of the load, and $f_{d}$ represents the disturbance force on the load.

The electrical equation of the electromagnet coil is [6,15,16]

(3) $$u=Ri+\frac{2k}{z}\dot{i}-\frac{2ki}{z^{2}}\dot{z},$$

where *u* is the port voltage of the electromagnet winding and *R* is the resistance of the electromagnet.

The feedback control is often applied to the port voltage of the electromagnet, that is

(4) $$u=U+k_{p}\left(z-z_{0}\right)+k_{d}\dot{z},$$

where *U* represents the port voltage in the static state, and $k_{p}$ and $k_{d}$ are, respectively, displacement and velocity feedback control coefficients.

By combining the above equations, we have

(5) $$\left\{\begin{array}{c} \dot{z}=v;\\ \dot{v}=g-k\frac{i^{2}}{mz^{2}}+\frac{f_{d}}{m};\\ \dot{i}=-\frac{z}{2k}Ri+i\frac{\dot{z}}{z}+\frac{z}{2k}\left(U+k_{p}\left(z-z_{0}\right)+k_{d}\dot{z}\right). \end{array}\right.$$

By adjusting the cont rol parameters $k_{p}$ and $k_{d}$, the system can maintain the stable suspension state.

## 3. Analysis of Vibration Characteristics

When the system is self-excited, the system often has a stable periodic solution near its equilibrium point [17,18]. To ensure the stability of the suspension control system, the above situation should be avoided [19,20,21].

Based on the above system equation, by ignoring the disturbance $f_{d}$, the equilibrium point of the system is solved. Let $\left(\dot{z},\dot{v},\dot{i}\right)=\left(0,0,0\right)$ and the isolated equilibrium point of the system *P* is

(6) $$P=\left(z_{0},v_{0},i0\right)=\left(i_{0}\sqrt{\frac{k}{mg}},0,\frac{U}{R}\right).$$

The Jacobian matrix of the system at the equilibrium point is [22]

(7) $$A=\left(\begin{array}{ccc} 0 & 1 & 0\\ \frac{2g}{z_{0}} & 0 & -\frac{2}{z_{0}}\sqrt{\frac{kg}{m}}\\ \frac{k_{p}z_{0}}{2k} & \frac{k_{d}z_{0}}{2k}+\sqrt{\frac{mg}{k}} & -\frac{Rz_{0}}{2k} \end{array}\right).$$

The characteristic equation of the matrix *A* is

(8) $$|\lambda I-A|=\lambda^{3}+\frac{Rz_{0}}{2k}\lambda^{2}+k_{d}\sqrt{\frac{g}{km}}\lambda +k_{p}\sqrt{\frac{g}{km}}-\frac{Rg}{k}.$$

The Routh table of the characteristic equation is shown as Table 1 [22,23].

According to the above Routh table, we can conclude the following:

(1) If the values of the first column of the Routh table are positive, namely $C_{11}>0,C_{21}>0,C_{31}>0$, $C_{41}>0$, all the characteristic roots of the matrix *A* have negative real parts. That is, corresponding to the nonlinear system in Equation Group (5), the linear system at equilibrium point *P* is stable [22] .

At this point, the matrix *A* has no pure virtual root, and the nonlinear system in Equation (5) is derivable in the neighborhood of the point *P*. The nonlinear system in Equation (5) and its corresponding linear system in Equation (7) in the equilibrium point *P* have the same topological structure, namely the nonlinear system in Equation (5) is also stable at the equilibrium point *P*.

(2) It is obvious that $C_{11}>0,C_{21}>0$. When $C_{31}=0$ or $C_{41}=0$, the matrix *A* has a characteristic root with negative real part, and the other two are pure virtual roots. That is, the linear system in Equation (7) is critical stable [22].

At this point, the nonlinear system in Equation (5) may exist the bifurcation with $C_{31}=0,C_{41}=0$.

In Case (2), the nonlinear system in Equation (5) may appear stable periodic solution, namely may induce the stable self-excited vibration.

When $c_{41}=0$, $k_{p}=R\sqrt{\frac{mg}{k}}$. As R,m,g,k are constant, $k_{p}$ should also be constant. In fact, the control parameter $k_{p}$ is constantly changing, and it is quite a coincidence that the above equation is met. Thus, the main discussion is the case $c_{31}=0$.

When $c_{31}=0$, one of the characteristic roots of the matrix *A* is a negative real root, and the other two are pure virtual roots. Let us assume that this pair pure virtual root is λ=a±ib, and the other negative real root is λ=d. Obviously, a=0.

Then, we have [24]

(9) $$\left(\lambda -a+ib\right)\left(\lambda -a-ib\right)\left(\lambda -d\right)=\lambda^{3}+\frac{Rz_{0}}{2k}\lambda^{2}+k_{d}\sqrt{\frac{g}{km}}\lambda +k_{p}\sqrt{\frac{g}{km}}-\frac{Rg}{k}.$$

Then,

(10) $$\left\{\begin{array}{c} -2a-d=\frac{Rz_{0}}{2k};\\ b^{2}+2ad=k_{d}\sqrt{\frac{g}{km}};\\ -b^{2}d=k_{p}\sqrt{\frac{g}{km}}-\frac{Rg}{k}. \end{array}\right.$$

According to Equation Group (10), we solve $a^{\prime }\left(k_{p},k_{d}\right)$. If only $a^{\prime }\left(k_{p},k_{d}\right)\neq 0$, we can get [20,22,25]

(11) $$-k_{p}+k_{d}+R\sqrt{\frac{mg}{k}}\neq 0.$$

Then, a Hopf bifurcation would occur at the point $c_{31}=0$ of the trust bearing system, which means that the system has the periodic solution at this point and a stable self-excited oscillation appears.

To achieve the stale suspension control and avoid the self-excited oscillation, according to $c_{11}>0,c_{21}>0,C_{31}>0,C_{41}>0$, we can also have

(12) $$R\sqrt{\frac{mg}{k}}<k_{p}<R\sqrt{\frac{mg}{k}}+\frac{k_{d}Rz_{0}}{2k}.$$

## 4. Design of the Adaptive Control Law

In the work process of the electromagnetic thrust bearing, the load mass changes very often. The inequality in Equation (12) would not hold if $k_{p}$ remains the same with the load mass variation, which will cause that the system may lose its stability or reach a critical steady state. Here, we design the adaptive controller parameters, that is the suitable selection of the suspension displacement coefficient $k_{p}$ and the differential feedback coefficient $k_{d}$, to make the above inequality hold.

In practical engineering, to make the inequality in Equation (12) exist, we let $k_{p}$

(13) $$k_{p}=R\sqrt{\frac{mg}{k}}+\frac{3Rz_{0}k_{d}}{10k}.$$

In the above equation, the load mass *m* is hard to measure directly. However, according to the previous analysis, at the isolated point *P* of the system, we have

(14) $$U_{0}=Ri_{0},mg=k\frac{i_{0}^{2}}{z_{0}^{2}},$$

where $U_{0}$ is the port voltage of the electromagnet at the equilibrium point. The port voltage *U* of the electromagnet coil is easy to measure.The adaptive control law with variable load mass is designed as follows [20,22,26]

(15) $$\left\{\begin{array}{c} k_{p}=\frac{U}{z_{0}}+\frac{3Rz_{0}k_{d0}}{10k}\sqrt{\frac{U}{U_{0}}};\\ k_{d}=k_{d0}\sqrt{\frac{U}{U_{0}}}, \end{array}\right.$$

where $k_{d0}$ is the velocity feedback coefficient of the equilibrium point.

## 5. Numerical Simulations

The system parameters of the thrust magnetic bearing system is given in Table 2.

(1) Stable suspension

**Figure 2:**
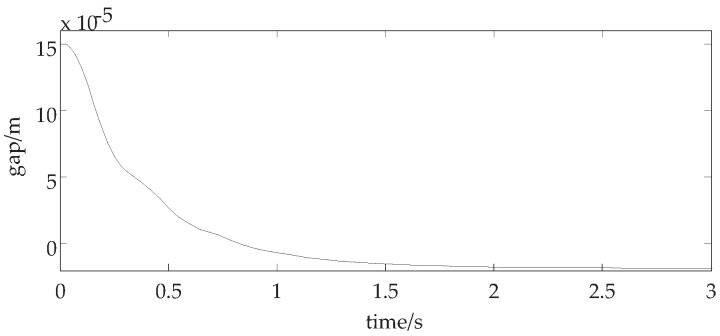
The suspension displacement curve with stable suspension.

By choosing $k_{p}=6700,k_{d}=60$, we can calculate $c_{31}>0,c_{41}>0$. Then, all the eigenvalues of the Jacobian matrix of the system have the negative real part, which means that the system can suspend stably without Hopf bifurcation and self oscillation. The suspension displacement curve with the above parameters is shown in Figure 2.

**Figure 3:**
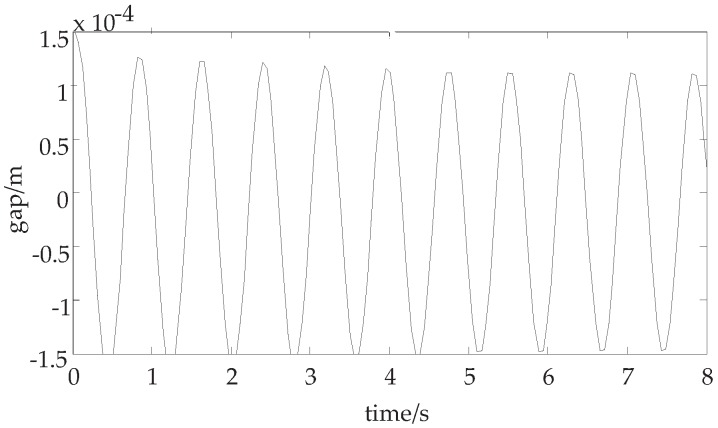
The suspension displacement curve with self-excited oscillation.

Figure 3 shows that, with the above parameters, the suspension gap reaches the equilibrium position 0 mm in the 1 s time, and then suspends stably without self-excited vibration.

(2) Self-existed oscillation

By choosing $k_{p}=7177,k_{d}=60$, we can calculate $c_{31}=0$. Therefore, the eigenvalues of the Jacobian matrix of the system has a pair of pure virtual root. The suspension displacement curve with the above parameters is shown in Figure 3.

Figure 2 shows that, with the above parameters, the suspension gap curve is in the periodic oscillation state and the obvious self-excited vibration occurs, which is consistent with the previous simulation analysis.

**Discussion 1.** In Figure 2 and Figure 3, when control parameters are changed, we discuss whether the system is stable. In Figure 2, the eigenvalues of the Jacobian matrix of the system have negative real part and the suspension displacement curve is convergent. While the control parameter kp is changed in Figure 3, the eigenvalues of the Jacobian matrix of the system have pure virtual part. The suspension displacement curve is divergent.

(3) The control parameters remain the same while the load mass changes

**Figure 4:**
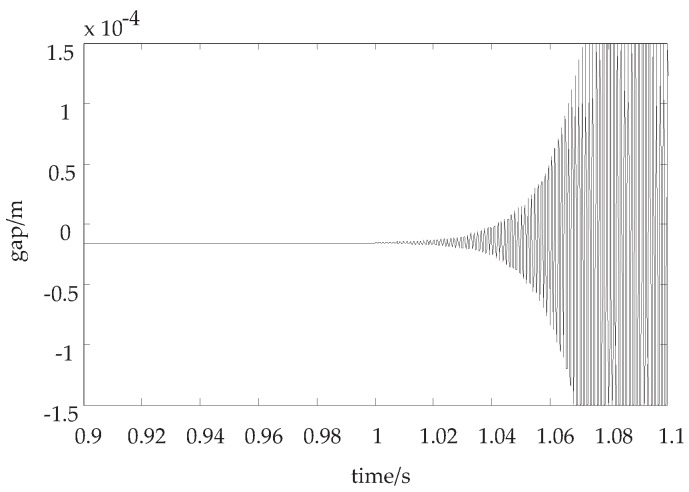
The suspension displacement curve while the load mass changes and the control parameters remain the same.

When the load mass changes up to 60 kg, the displacement curve is shown in Figure 4 while the control parameters remain the same.

As Figure 4 shows, while the load mass suddenly changes, with the same control parameters, the suspension displacement would oscillate and the amplitude would increase gradually, and the system will lose the stability eventually.

(4) Adopting the adaptive control law while the load mass changes

**Figure 5:**
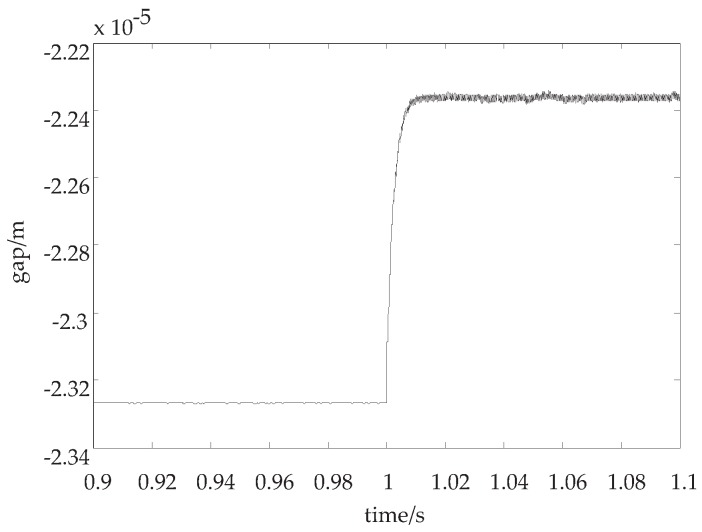
The suspension displacement curve with the adaptive control parameters while the load mass changes.

When he load mass changes up to 60 kg, the displacement curve of the system by adopting the adaptive control law is shown in Figure 5.

In Figure 5, when the suspension mass suddenly changes, the curve of the displacement gradually approaches the new equilibrium point, while it maintains a stable suspension state by adopting the adaptive control law.

**Discussion 2.** In Figure 4 and Figure 5, when the load mass is increased, we discuss whether the system is stable. In Figure 4, the control parameters keep unchanged, so the suspension displacement curve is divergent. However, if the adaptive control law is adopted, the suspension displacement curve is convergent in Figure 5.

## 6. Conclusions

Since a wide range of load mass variation of the electromagnetic thrust bearing would degrade the system performance significantly and even cause the self-excited vibration problem, we put forward a kind of adaptive control law which can tolerate this large range of load changes. By changing the nonlinear system as a class of linear systems, this paper analyzes the boundary conditions when the self-excited oscillation exists. The designed control law can adjust the controller parameters according to the change of the load mass, which makes the system away from self-excited oscillation area and possess a good stability margin.

## Figures and Tables

**Table 1:** Routh table of the characteristic equation of the matrix *A*.

$\lambda^{n}\left(n=0,1,2,3\right)$	$C_{i1}\left(i=1,2,3,4\right)$	$C_{j2}\left(j=1,2,3,4\right)$
$\lambda^{3}$	$C_{11}=1$	$C_{12}=k_{d}\sqrt{\frac{g}{km}}$
$\lambda^{2}$	$C_{21}=\frac{Rz_{0}}{2k}$	$C_{22}=k_{p}\sqrt{\frac{g}{km}}-\frac{Rg}{k}$
$\lambda^{1}$	$C_{31}=\frac{2mg}{kz_{0}}+k_{d}\sqrt{\frac{mg}{k^{3}}}-\frac{2k_{p}}{Rz_{0}}\sqrt{\frac{mg}{k}}$	
$\lambda^{0}$	$c_{41}=k_{p}\sqrt{\frac{g}{km}}-\frac{Rg}{k}$	

**Table 2:** Parameter values of the electromagnetic thrust bearing system.

Name	*m*	*g*	*k*	*R*	$i_{0}$	$z_{0}$
Value	40	9.8	$8.82\times 10^{-6}$	1	1	0.00015
Unit	kg	$m/s^{2}$	$N\times m^{2}/A^{2}$	Ω	A	m

## References

[1] Hijikata K., Kobayashi S., Takemoto M., Tanaka Y., Chiba A., Fukao T. (2008). Basic Characteristics of an Active Thrust Magnetic Bearing With a Cylindrical Rotor Core. IEEE Trans. Magn..

[2] Balachandran B., Kalmár-Nagy T., Gilsinn D.E. (2009). Delay Diffrential Equation: Recent Advances and New Directions.

[3] Gakkhar S., Negi K., Sahani S.K. (2009). Effects of seasonal growth on ratio dependent delayed prey-predator system. Commun. Nonlinear Sci. Numer. Simul..

[4] Gakkhar S., Sahani S.K., Negi K. (2009). Effects of seasonal growth on delayed prey-predator Model. Chaos Solitons Fractals.

[5] Guo S., Chen Y., Wu J. (2008). Two-parameter bifurcations in a network of two neurons with multiple delays. J. Differ. Equ..

[6] Zhang Z.Z., Li X.L. (2018). Real-time adaptive control of a magnetic levitation system with a large range of load disturbance. Sensors.

[7] Zhang L.L., Zhang Z.Z., Huang L.H. (2012). Double Hopf bifurcation of time-delayed feedback control for maglev system. Nonlinear Dyn..

[8] Hassard B., Kazarinoff N., Wan Y. (1981). Theory and Applications of Hopf Bifurcation.

[9] Huang C., He Y., Huang L., Yuan C. (2008). Hopf bifurcation analysis of two neurons with three delays. Nonlinear Anal..

[10] Ho Y.S., Liu H., Yu L. (2003). Effect of thrust magnetic bearing on stability and bifurcation of a flexible rotor active magnetic bearing eystem. J. Vib. Acoust. Trans. ASME.

[11] Bélair J., Campbell S.A. (1994). Stability and bifurcations of equilibria in a multiple-delayed differential equation. SIAM J. Appl. Math..

[12] Sanagawa Y., Ueda H., Tsuda M., Lshiyama A. (2001). Characteristics of lift and restoring force in HTS bulk-Application to two-dimensional maglev transporter. IEEE Trans. Appl. Supercond..

[13] Sunita G., Anuraj S. (2012). Complex dynamics in a prey predator system with multiple delays. Commun. Nonlinear Sci. Numer. Simul..

[14] Wang H.P., Li J., Zhang K. (2007). Stability and Hopf bifurcation of the maglev system with delayed speed feedback control. Acta Autom. Sin..

[15] Santos M.P.S.D., Ferreira J.A.F., Simões J.A.O., Pascoal R., Torrão J., Xue X.Z., Furlani E.P. (2016). Magnetic levitation-based electromagnetic energy harvesting: a semi-analytical non-linear model for energy transduction. Sci. Rep..

[16] Hong S.K., Langari R. (2000). Robust fuzzy control of a magnetic bearing system subject to harmonic disturbances. IEEE Trans. Control. Syst. Technol..

[17] Whidborne J.F. (1993). EMS control system design for a maglev vehicle-A critical system. Automatica.

[18] Zhang Z., Zhang L. (2013). Hopf bifurcation of time-delayed feedback control for maglev system with flexible guideway. Appl. Math. Comput..

[19] Xiao J.Z., Jian J.W., Zhou Y.H. (2005). Effect of spring non-linearity on dynamic stability of a controlled maglev vehicle and its guideway system. J. Sound Vib..

[20] Yuan S., Song Y. (2009). Bifurcation and stability analysis for a delayed Leslie Gower predator-prey system. IMA J. Appl. Math..

[21] Zhang J.Y., Yang Y.R., Zeng J. (2000). An algorithm criterion for Hopf bifurcation and its applications in vehicle dynamics. Chin. J. Theor. Appl. Mech..

[22] She L.H., Liu G.D., Shi X.H. (2006). Adaptive control of maglev system according to HOPF bifurcation. J. Dyn. Control..

[23] Zhang L., Huang L., Zhang Z. (2009). Stability and Hopf bifurcation of the maglev system with delayed position and speed feedback control. Nonlinear Dyn..

[24] Zhang L.L., Campbell S.A., Huang L.H. (2011). Nonlinear analysis of a maglev system with time-delayed feedback control. Phys. Dyn..

[25] Hale J.K. (1993). Introduction to Functional Differential Equations.

[26] Zheng J., Deng Z.G., Wang L.L., Liu L., Zhang Y., Wang S., Wang J. (2007). Stability of the Maglev Vehicle Model Using Bulk High TC Superconductors at Low Speed. IEEE Trans. Appl. Supercond..

